# Supplementary material for: Hematologic profile of Amazon river dolphins Inia geoffrensis and its variation during acute capture stress
Source: PLoS One. 2019 Dec 26;14(12):e0226955. doi: 10.1371/journal.pone.0226955 (PMC6932813; doi:10.1371/journal.pone.0226955)
Supplement: Attachment — Submitted filename: PONE-D-19-17847 Review.docx [file pone.0226955.s002.docx]

**Review of “Hematologic profile of Amazon river dolphins Inia geoffrensis and its variation during acute capture stress”**

Overall Comment:

The data presented here represent important contributions to the literature regarding blood reference ranges for the understudied boto population. I do have some reservations regarding its publication at this time, and request a revision for the following reasons.

I am concerned about the inclusion of animals with high stress scores in the creation of baseline values/reference ranges. Ideally, animals with high stress scores would be excluded from the reference range creation since they are not “normal.” I see this paper as having two separate purposes, 1) to establish hematological reference ranges for minimally stressed boto by age class and 2) to evaluate the effect of stress on hematological parameters in this species. I think it would be a stronger manuscript if organized as such. If you choose not to split the study out in this manner, there should be some mention of this as a study limitation in the discussion (the inclusion of stressed animals when establishing baselines).

I was unclear whether or not all the data were tested for a normal or Gaussian distribution. Especially for some of the parameters with relatively low sample sizes, this is extremely important with regards to the question on whether rigorous statistical methods were applied. I answered “I don’t know” because this is a pending question I have for the authors in my review.

While the authors in general did a good job of translating to English, the language is still awkward in many places and there are still some confusing statements and grammatical errors that appear to be associated with translation. I tried to assist and interpret where I could, but I recommend a native English speaker edit the manuscript prior to resubmission.

Abstract:

Line 30, 36: Change “gender” to “sex” throughout text as sex refers to the biological differences between male and female, and gender refers to the individual’s identity and is therefore not suitable for use with animals.

Lines 36-40: Since reference ranges are often created according to sex, reproductive status, and age class, I would recommend moving these findings up to line 33 before the sentence starting “Means…” You have essentially created age-class specific reference ranges in this study since those were the groups with statistically significant differences – and I would clarify that in the abstract.

Lines 38-39: I recommend deleting “as a result of the still-developing immune system.” There are other potential explanations for calves having higher WBCs (higher lung worm burden in younger animals - which has been observed in other dolphin populations, or higher stress level than older animals). Given these alternate possible explanations and the fact that this is not a common finding in all populations of dolphins, I do not recommend being so definitive in explaining its cause.

Introduction:

Line 52: Change “accessed” to “assessed”

Line 55: Recommend changing “health status approaches” to “health assessments”

Line 56-57: All these studies can be referred to as “capture-release” studies, I am unclear why the distinction is being made for the St. Aubin study as “netted wild population” vs capture release programs. Please clarify.

Line 57: Change semicolon to comma and “parameters” to “parameter” and “baseline” to “baselines”

Lines 58-62: This sentence is confusing the way it is written, please reword. With reference #11 – there had not been a baseline study prior to the oil spill on that particular population (although baseline studies existed for other Tt populations that were used for comparison). I would be more explicit in explaining how this particular citation makes the authors’ point. I also think the reference to seasonal variation in the Sarasota dolphins is confusing since you do not address seasonality at all in the remainder of the paper (even though it is controlled for with your sample timing).

Line 64: delete “aged”

Line 65: This is not necessarily a common finding among the capture-release sampled dolphins in various locations. Given the age of this citation, I would recommend either striking this comment or finding additional more recent citations to make a stronger case.

Line 67: Change “alteration on” to alterations in”

Line 69: change “englobe” to “involve”

Line 70: change “may reflect” to “may be reflected in”

Line 71: change “gender” to “sex”

Line 73: I believe you mean “before establishing baseline blood parameters.” Ideally you are establishing these reference ranges with animals that are minimally stressed and removing the outliers from your “normal” sampling population.

Line 76: include citation for the most recent IUCN report

Line 76-77: Recommend changing “under intentional catch” (which is vague) to “actively fished commercially”

Line 78: Recommend changing “leading to significant populations decrease” to “leading to a significant population decline”

Lines 83-84: delete comma after “reserve” and replace with “in the”

Line 87: Why single out the ESR here? Seems unnecessary – either please justify or delete.

Materials and Methods:

Line 109: Recommend spelling out the RDSM acronym in the figure caption

Line 116: Change “proximities of research floating station” to “proximity of the floating research station”

Lines 116-117: Recommend changing the sentence starting with “It varied” to: “The number and behavior of encircled animals varied according to environmental conditions.”

Line 118: If you are going to indicate the depth of the channel it would also be helpful to know the depth of your capture net (s).

Lines 119-121: You state that the usual method was to seine the dolphins to the shoreline. That leads me to believe that there were other methods used to capture the dolphins in some instances. If so, please describe those methods as well, if not, please clarify the text here.

Line 126: What were your criteria for determining pregnancy? Identification of a fetus? Recommend explicitly stating that.

Line 139: Did you actually use a catheter or a needle? Was the blood drawn directly into the EDTA vacutainer tube or was it drawn into a syringe and then transferred to the tubes? The way this is written, it sounds like the blood from the EDTA tubes was transferred to a tube with no anticoagulant and allowed to clot, which is confusing. Please clarify your methods.

Line 146: How were these manual methods standardized? Were they consistently done by a single trained individual or were multiple individuals trained on standardized methods?

Line 171: For stress levels 1 & 2 behavior, perhaps you mean no/little excessive response to external stimuli? Or appropriate response to external stimuli? Relaxed dolphins should be alert and responsive to external stimuli, but not hyper-responsive. By immobilization do you mean restraint? Recommend re-wording these behavioral descriptions.

Line 175: I think you mean “the neutrophil to lymphocyte ratio” ?

Lines 176-177: 12 individuals in each category? Are samples from the same individual compared between the two time categories or are the comparisons done between different animals? Please clarify the methods here.

Line 177: insert ‘of’ between ‘chronology’ and ‘acute’

Line 181: Recommend first discussing the tests for significant differences in age class, reproductive status, and sex, then discuss the methods for creating reference ranges based on those outcomes. Were all the data tested for normality (Gaussian distribution)? Especially with some of the parameters that had smaller sample size, normal distribution cannot be assumed and should be tested.

Line 200: Strength of correlation (weak, moderate, or strong according to r value) should be stated here. You can find references in the literature to describe correlation strength.

Line 195: How were multiple comparisons controlled for in order to prevent Type I error (false positives)? You compared a lot of variables and should either control for these multiple comparisons or justify why you did not do so.

Line 214: Since Table 4 is cut off, I cannot see if you stated 90% RI for the calves. I am assuming you did not due to small sample size. I would explicitly state what you are presenting for the calf data here and why there are not reference ranges established. I would also recommend removing the moderate and severely stressed dolphins from the reference range calculation.

Line 220: Formatting in the version I have cuts off the left (parameter) and right (last column is calves min) margins of this table. Cannot fully evaluate data presented. Recommend including more details regarding the reference values in the table caption (90% double sided reference intervals)

Line 221: In the methods, it stated the level of significance was set at p < 0.05 but here it states p < 0.001. Why the discrepancy?

Line 234: In Fig 2: Recommend adding sample sizes (n) above each age class box plot

Line 242: Again, recommend including sample sizes in Fig 3 (either in the caption or in the legend)

Line 251: I do not see medians in table S1, delete reference to them here.

Line 252: These are not reference intervals, recommend deleting ”RI” and just referring to the data in table S1 as summary statistics.

Line 263: Fig 4: Recommend including r and p values either on the figure or in the caption. Same comment for Figs 5 and 6 as well.

Line 254: Recommend spelling out cardiac rate and respiratory frequency in this section title as these are not common abbreviations.

Line 268: Correlation should be categorized as weak, moderate or strong based on the r values.

Discussion:

Line 300: change “comes” to “come” – “data” is a pleural noun (singular “datum”)

Line 301: change to “in the 1970’s”

Lines 304-310: Captive boto were also likely less stressed than the wild-caught boto, which would also affect the WBC count. Subclinical bacterial, fungal, protozoal, and viral infections should also be included in the differentials for elevated WBC in wild vs managed populations.

Line 320: change “an” to “a” Also, without demonstrating a correlation between ESR and inflammation, I would caution against evaluating this parameters utility here. While the narrow range MAY indicate that this could be a useful indicator IF it does in fact change with inflammation, you have not proven the latter yet.

Lines 318-320: Was there any correlation between ESR and WBC count? If not, this makes it more likely that the WBC/neut elevations were due to stress rather than inflammation/infection/antigenic stimulation.

Lines 321-330: Also need to include stress as a differential for higher WBC counts in juveniles. And a note that this is not a consistent finding among all cetacean populations.

Line 335: change “was” to “were”

Line 340: Change “towards” to “on”

Line 342: Can you present data in the results regarding the sample sizes according to each trimester if available to help elucidate this point?

Line 343: change “values” to “value” and “towards non-pregnant females” to “between pregnant and non-pregnant females”

Lines 356-358: Due to the spleen’s small size relative to the dolphin’s overall body mass, my understanding is that unlike in other species, splenic contraction is not considered a significant differential for elevated RBC, HCT, Hb in cetaceans.

Line 359: Recommend using the term “epinephrine” rather than “adrenalin”

Line 369: change “access” to “assess”

Line 373-374: Also cannot rule out subclinical pulmonary disease as contributing to this trend, and this should be included as a differential for that finding.

Lines 376-378: Please reword this sentence for clarity.

Line 379: Add “can” between ‘animals’ and ‘have’ – as this is not always a consistent finding in cetaceans

Lines 384-394: More clarification in your discussion of the different types of stress leukograms is warranted – since you have the expected neutrophilia with a corticosteroid stress response, but not the associated lymphopenia (as with the “typical stress leukogram” that you cite). Lymphocytosis would be associated with an epinephrine-induced response. While you mention both of these hormone-induced pathways, your explanation of the lymphocytosis is incomplete.

Line 400: change ‘access’ to ‘assess’

Line 403: change “ad” to “and”
