# Supplementary material for: Hematologic profile of Amazon river dolphins Inia geoffrensis and its variation during acute capture stress
Source: PLoS One. 2019 Dec 26;14(12):e0226955. doi: 10.1371/journal.pone.0226955 (PMC6932813; doi:10.1371/journal.pone.0226955)
Supplement: Attachment — Submitted filename: PONE-D-19-17847 Review.docx [file pone.0226955.s003.docx]

PONE-D-19-17847

Hematological profile of Amazon river dolphins *Inia geoffrensis* and its variation during acute capture stress.

de Mello & da Silva

- The title identifies the study subject as Amazon river dolphin, also known as boto. But the interchange of this common name in the manuscript may be confusing to the reader. I would recommend being consistent in using one. Mention both names once, but after that be consistent in using one.
- In Spanish and perhaps in Portuguese, the names of rivers are begin with capital letters, but the word river itself does not begin with capital letters, i.e., río Amazonas. But in English, both the river name and the word river begin with capital letters. Thus, in the title and throughout the document, it should be Amazon River dolphin.
- All animals, except common animals (horses, goats, swine, cattle), should be first introduced with a common name followed by its scientific name. Afterwards, I recommend using just the common name.
- Add the common names for Globicephala macrorhynchus, Pseudorca crassidens, Orcinus orca, Delphinapterus leucas, Stenella coeruleoalba
- Add the scientific names for Amazonian manatee, rhinoceros

Lines:

31: 110 write out as One-hundred-and-ten

38: Delete The before calves

40: should be lymphocytes

42: should be neutrophils, lymphocytes and monocytes

44: define Hct and then put in parenthesis. Red Blood Cells should be red blood cells. Define Hb and then put in parenthesis.

65: should be white blood cells (WBC)

66: should be hematocrit (Hct)

74: should be The Amazon river dolphin or boto (*Inia geoffrensis*)

76: cite where the IUCN lists the species as endangered.

76: instead of “under intentional catch” use affected by intentional capture

81: delete or boto (I. geoffrensis)

83: should be Mamirauá Sustainable Development Reserve (RDSM) and Amazon River.

103: delete Mamirauá Sustainable Development Reserve and delete the parenthesis in RDSM

144: Comment: the red top tubes were allowed to clot at ambient temperature, room temperature or in the refrigerator? Please specify.

147: delete white blood cell and delete delete the parenthesis in WBC

167: should be Cardiac rate (CR, beats/minute)

168: should be respiratory frequency (RF, breaths/minute)

174: Should be (Table 3)

178: should be CR and RF were

202: 110 write out as One-hundred-and-ten

202-203: Delete by Projeto Boto,

214-217: box plot and histogram analyses (Table 4). Then delete line 215, 216, 217.

231: change white blood cells counts for WBC

234: Delete Blood cell (and the other ).

270: cardiac rate should be CR, and respiratory frequency should be RF

277: understandably/expected should be understandably and expected

279: cadiac rate and respiratory frequency should be CR and RF

280: put a comma (,) after (p=0.003)

282: respiratory frequency should be RF

283: respiratory frequency should be RF

287: respiratory frequency should be RF

288: cadiac rate should be CR

301: by the 1970s. It does not 1970’s because it is not possessive. It is plural, so it should be 1970s

305: Where it says I. geoffrensis, change for ones

317: use common name before Globicephala macrorhynchus and Pseudorca crassidens

327: use common names for Orcinus orca, T. truncatus, Delphinapturus leucas in conjunction with scientific name

356: Hb instead of hemoglobin

362: the scientific name for the pantropical spotted dolphin is Stenella atteanuata. Stenella tropicalis does not exist.

373: should be RF for respiratory frequency

375, 385, 386, 391: Explain first what is N:L before using it.

387: follow the scientific name for Amazonian manatee

388: rhinos is a popular way to refer to rhinoceros. However, which species of rhinoceros are you referring to? White rhinoceros (*Ceratotherium simum*), black rhinoceros (*Diceros bicornis*), Indian rhinoceros (*Rhinoceros unicornis*), Javan rhinoceros (*Rhinoceros sondaicus*) or Sumatran rhinoceros (*Dicerorhinus sumatrensis*). Or is it to species of the Rhinocerotidae?

389: common name for Stenella coeruleoalba

397: instead of boto, use Amazon River dolphin

401: use Hct and Hb

403: use CR and RF. The and is missing an n.

Figure 1: Legends inside figure should be in English or the same terms used in the text.

Very good clinical work, and much needed for this species.

Congratulations in this accomplishment.
